# Optimization of the extraction of the *p*‐menthadienol isomers and aristolone contained in the essential oil from *Elyonurus hensii* using a 2^3^ full factorial design

**DOI:** 10.1002/fsn3.459

**Published:** 2017-02-09

**Authors:** Aubin Nestor Loumouamou, Kévin Bikindou, Ernest Bitemou, Pierre Chalard, Thomas Silou, Gilles Figueredo

**Affiliations:** ^1^ Equipe Pluridisciplinaire de Recherche en Alimentation et Nutrition (EPRAN)Faculté des Sciences et des TechniquesUniversité Marien NgouabiBrazzavilleCongo; ^2^ Institut National de Recherche en Sciences Exactes et Naturelles (IRSEN)BrazzavilleCongo; ^3^ SIGMA ClermontUniversité Clermont AuvergneClermont‐FerrandFrance; ^4^ Institut de Chimie de Clermont‐FerrandCNRS, UMR 6296AubièreFrance; ^5^ Ecole Supérieure de Technologie (EST‐Cataractes)BrazzavilleCongo; ^6^ Laboratoire d'analyse des Extraits végétaux et des Arômes (LEXVA)Biopole, Clermont‐Limagne, Saint‐BeauzireFrance

**Keywords:** 8‐dien‐1‐ol, 8‐dien‐2‐ol, aristolone, *cis* and *trans‐p*‐mentha‐1(7), *cis* and *trans‐p*‐mentha‐2, *Elyonurus hensii*, modeling

## Abstract

The aim of this study was to optimize the extraction of *p*‐menthadienol isomers and aristolone from the essential oil of *Elyonurus hensii* by hydrodistillation. The study of the seasonal variation in the chemical composition has shown that the plant material has been subject to a natural selection regarding the biosynthesis of the *p*‐menthadienol isomers: during periods of water stress, the extracts are rich in *cis* and *trans‐p*‐mentha‐1(7),8‐dien‐2‐ol and poor in *cis* and *trans‐p*‐mentha‐2,8‐dien‐1‐ol. Regarding the modeling, eight experiments were carried out by considering three easily interpretable factors (the extraction duration, the residual water content and the state of the division of the plant material). The average yield was 1.33% for the aerial part and 0.74% for the roots. The residual water content is the most important factor, which significantly influences the average yield of the essential oil and the content of the major constituents. Regarding the aerial part, a low residual water content of the plant material varies the essential oil yield (from 0.40% to 2.11%) and the content of *cis* and *trans*‐*p*‐mentha‐2.8‐dien‐1‐ol (from 15.87% to 23.24%). At the root level, the samples that have a very low residual water content provide extracts richer in aristolone. The combined effects of the extraction duration, the state of division, and the residual water content influence greatly the extraction of aristolone (from 36.68% to 54.55%). However, these interactions are more complex and difficult to assess.

## 1 Introduction

*Elyonurus hensii* is found in the tropical and subtropical regions of South America (Brazil and Argentina), Africa (Congo Republic, Gabon, DR Congo, Angola), and Australia (Yang et al., 2013). *Elyonurus hensii* has been studied in only the Republic of Congo, where this plant grows spontaneously on the “Plateau des Cataractes”. It is usually used by local people as a théiforme drink to relieve pain, which justifies its vernacular name “tikoni” (pain).

The first description of the chemical profile of the essential oils from different parts (roots, stems, leaves, and flowers) showed that the oils from the aerial parts were mainly rich in *p‐*menthadienol isomers, and the main ones are *cis* and *trans‐p*‐mentha‐1(7),8‐dien‐2‐ol, *cis* and *trans‐p*‐mentha‐2,8‐dien‐1‐ol. The essential oil of the roots is rather rich in sesquiterpene compounds, and aristolone, with a content of approximately 40%, is the major compound. (Silou, Loubaki, Figuérédo, & Chalchat, 2006). Studies of the essential oils of species of the genus *Elyonurus* are very limited; however, it appears that *Elyonurus hensii* is the only species of the genus that is rich in *p‐*menthadienol isomers. However, these isomers are present at significant contents in the oils of *Cymbopogon giganteus* and *Cymbopogon densiflorus*. Indeed, the essential oil of *Cymbopogon giganteus* from the Ivory Coast is characterized by high contents of *cis* and *trans‐p*‐mentha‐2,8‐dien‐1‐ol (8.7% and 18,4%, respectively) and *cis* and *trans‐p*‐mentha‐1(7),8‐dien‐2‐ol (16% and 15.7%, respectively), and this oil presents antimicrobial properties (Boti et al., 2006). Moreover, the antimicrobial properties were also observed on the essential oil of *Cymbopogon giganteus* from Burkina Faso (Menut et al., 2000), Cameroon (Jirovetz, Buchbauer, Eller, Ngassoum, & Maponmetsem, 2007), Benin (Ayedoun, Sohounhloué, Menut, Lamaty, & Bessière, 1999), and Mali (Sidibe, Chalchat, Garry, & Hamara, 2001); these species produce essential oils rich in *p*‐menthadienol isomers. The essential oil from the flowers and leaves of *Cymbopogon densiflorus* of Zambia is also rich in *p‐*menthadienol isomers, and it contains 22.4% *trans‐p*‐mentha‐2,8‐dien‐1‐ol and 11.1% *cis‐p*‐mentha‐1(7),8‐dien‐2‐ol. The oil of the Brazilian plant presents a similar composition (Boelens, 1994; Chisowa, 1997). The literature reports several uses of *Cymbopogon densiflorus*: the crushed leaves are used as a treatment for rheumatism in Gabon, the flower head is smoked in a pipe as a cure for bronchial affections in Malawi, and the plant sap is used in the Congo‐Brazzaville, where it is also given as a treatment for asthma and to calm fits (Akhila, 2010). Manifestly, the essential oil of *Cymbopogon densiflorus* exhibits biological activities. The massive presence of *p‐*menthadienol isomers in the essential oil of *Elyonurus hensii* points also to possible antimicrobial properties. Aristolone is also an interesting constituent since it is likely to induce an antalgic activity (Tian‐Shung, Amooru, Damu, & Kuo, 2004).

In a previous study of *Elyonurus hensii* from Congo‐Brazzaville, (1) the volatile components from the stems and the roots were obtained by hydrodistillation and head‐space solid phase microextraction (HS‐SPME), and (2) for the extracts obtained with methanol, ethyl acetate and dichloromethane, the antioxidant activities of the extracts were demonstrated and compared. The essential oil of *Elyonurus hensii* did not have significant properties of DPPH and ABTS^.+^ for either the stems or the roots. However, the solvent extracts are effective antioxidants according to in vitro assays. The authors conclude that the extracts of *Elyonurus hensii* have potential as natural additives for the food and pharmaceutical industries (Yang et al., 2013).

The use of experimental designs can improve the yields of essential oil. They also optimize the extraction of the major constituents of the essential oils by organizing the steam distillation with a minimum number of experiments to be performed (Silou, Malanda, & Loubaki, 2004; Silou et al., 2009). Taking into account the scientific interest that seems to represent the isomers of *p*‐menthadienol and aristolone, we have tried to optimize the extraction of these major constituents by using a 2^n^ full factorial design. We have used a simple model of the first degree which gives the representation of the response function based on variables, which allows us to evaluate the influence of different factors studied on the contents of the aristolone and *p*‐menthadienol isomers.

## 2 Material and Methods

### 2.1 Plant material

The plant material consisting of the aerial parts (stems, leaves, and flowering tops) and the roots was harvested. The samples were collected on the “plateau des cataractes” (District of Louingui, Pool Department, Republic of Congo) at three sites, Loufoulacari (L), Campus rural (C), and Sese (S), and during different period of the year 2015, february (f), may (m), july (j), november (n), and december (d). These harvest periods include periods of drastic reductions in rainfall, the dry season (may and june), and periods of heavy rainfall, the rainy season (november and december). The precipitation levels on the “plateau des Cataractes” are an average of 2 mm in the dry season and 250 mm in the rainy season. Moreover, the sites of Loufoulakari and rural Campus are on a clay ground, whereas the Sese site is located on a sandy soil.

For the determination of the chemical composition, the plant material was dried in the shade for 8 days and then subjected to steam distillation. Indeed, from the sixth day, the loss of water reached a plateau, as is the case for many species of the Poaceae family (Silou et al., 2004).

### 2.2 Extraction of the essential oil

After drying the plant material, the essential oil has been obtained by steam distillation using a Clevenger‐type apparatus (Clevenger, 1928). The heating temperature was fixed at 100°C, which allowed a constant flow of condensation. Each time, 300 g of vegetable material, consisting either of roots or stems, leaves, and young flowers was placed in a flask with 500 ml of water and subjected to distillation for 3 hr. The organic phase from the distillation was separated from the aqueous phase by extraction with diethyl ether. The organic phase obtained was then dried over anhydrous sodium sulfate to remove traces of water, and the essential oil was recovered after the evaporation of the diethyl ether on a rotary evaporator. The samples of essential oil were stored in a refrigerator before submitting them to GC‐FID and GC‐MS analysis.

### 2.3 Determination of the chemical composition

#### 2.3.1 Analysis by gas chromatography (GC)

The quantitative analysis of the essential oil was carried out using an Agilent gas chromatograph model 6,890 equipped with a DB5 MS column (20 m × 0.18 mm; 0.18 μm). The oven temperature was programmed to 50°C for 3.2 min, then heated to 300°C at a rate of 10°C/min. The temperatures of the injector and the flame ionization detector (FID) were maintained at 280°C. The essential oils were diluted in acetone to 3.5% (v/v) and injected in split mode (1/60); hydrogen was used as the carrier gas (1 ml/min), and the injection volume was 1 μl. At the same time, a solution of *n*‐alkanes (C8‐C30) was analyzed under the same conditions to calculate retention indices (RI) using the Van den Dool and Kratz equation (Van Del Dool & Kratz, 1963). The relative concentrations of the compounds were calculated from the peak area obtained by gas chromatography without using correction factors.

#### 2.3.2 Analysis by coupling gas chromatography and mass spectrometry (GC‐ MS)

Qualitative analysis was performed using an Agilent gas chromatograph model 7,890 coupled to a Agilent mass spectrometer model 5975 equipped with a DB5 MS column (20 m × 0.18 mm; 0.18 μm). The oven temperature was 50°C and remained constant for 3.2 min; then, it was increased to 300°C at a rate of 8°C/min. The injector temperature was 280°C. Ionization was obtained by electron impact at 70 eV, and the electron multiplier was maintained at 2200 eV. The temperature of the ion source was 230°C. Mass spectral data were acquired in the scan mode in the range m/z 33–450. The flow of carrier gas (helium) was set at 0.9 ml/min; compound identification was made by comparison of their spectra and RI with those of libraries such as Adams (2008), Nist (2008), and Köning, Hochmuth, and Joulain (2001) and were incorporated in the laboratory.

### 2.4 Modeling of essential oil extraction by hydro distillation

The variables influencing extraction yield were as follows: time, temperature, condensation rate, the state of division of the plant material, the mass ratio of the plant material to water, and water loss from the plant material (Denny, 1991). A model with six variables, even in the case of a first‐degree model, would require 2^6^ = 64 experiments (Goupy, 2001). For experimental convenience, some variables were thus kept constant. We considered three variables: the extraction duration (X_1_), the residual water content (X_2_), and the state of the division of the plant material (X_3_). These three factors offered the advantage of being easy to control. Extraction yield (y), *p*‐menthadienol isomers, and aristolone contents (Z) depend on factors X_1_, X_2_, and X_3_. Mathematically, this is expressed as y or z = f(X_1_, X_2_, X_3_), where y and z are the responses, f(X) is the response function, and X_1_, X_2_, and X_3_ are the factors taken into account. The experiment is designed to determine the effects of certain factors on each response.

The two‐level factorial design as developed by Davies (1954) is well‐suited to addressing this type of question (Ortigosa, 1993). The general formula for a complete factorial plan with N experiments is *N* = 2^k^, where k is the number of variables in the factorial. If k = 3, then *N* = 2^k^ = 2^3^ = 8 experiments.

To construct the experiment matrix, we define reduced variables x_i_ as the following:

x_i_ = (X_i_ – X_0_)/ΔX; X_0_ is the base value at the centre of the experimental domain (level 0), and ΔX is the variation step, that is, the unit of variation in the variables. Table 1 gives the two levels of the variables in the steam extraction of *Elyonurus hensii*.

**Table 1:** Levels of the variables in the steam extraction of *Elyonurus hensii*

Level	Extraction duration (hr) X_1_	Residual water content^a^ X_2_	State of division (cm) X_3_
Low (*−*1)	1.5	Low	<5
High (+1)	3	High	10

a The residual water content is low when the distillation is carried out with the plant material dried during 8 days and high when the fresh material is distilled. For the aerial part, the residual content varies from 62% (high) to 4% (low); for the roots, it varies from 35% to 6%.

The domain of the study with coded variables becomes the domain (−1; +1), and the eight responses described by the experimental matrix are set up after randomization. The combination of these three variables and the two levels by variable lead to the following experimental design (Table 2).

**Table 2:** Experimental matrix for essential oil extraction from *Elyonurus hensii*

Run	X_1_	X_2_	X_3_	Responses	
				Y	Z
1	−1	−1	−1	Y_1_	Z_1_
2	+1	−1	−1	Y_2_	Z_2_
3	−1	+1	−1	Y_3_	Z_3_
4	+1	+1	−1	Y_4_	Z_4_
5	−1	−1	+1	Y_5_	Z_5_
6	+1	−1	+1	Y_6_	Z_6_
7	−1	+1	+1	Y_7_	Z_7_
8	+1	+1	+1	Y_8_	Z_8_
Level ‐1	1.5	low	<5		
Level +1	3	high	10		

For a first‐degree model with interactions, the representative points of a three‐variable experimental design are located in three‐dimensional space. The corresponding response function is a first‐degree polynomial for each factor taken separately. It is notated as the following:

$$y=a_{0}+a_{1}x_{1}+a_{2}x_{2}+a_{3}x_{3}+a_{12}x_{1}x_{2}+a_{13}x_{1}x_{3}+a_{23}x_{2}x_{3}+a_{123}x_{1}x_{2}x_{3}$$

If the mathematical model associated with the factorial plan is constructed with centered, reduced variables, the coefficients of the polynomial thus have very simple meanings: average *a*_0_, main effects *a*_*i*_, and interactions *a*_*ij*_ and *a*_*ijk*_ (Goupy, 2001).

## 3 Results

### 3.1 Chemical composition

The essential oil of the aerial part (stems, leaves, and flowering tops) obtained with the samples collected from the Loufoulakari site is mainly rich in oxygenated monoterpenes whose contents range between 50.81% and 71.59%, with the following major compounds the isomers of *p*‐menthadienol: *trans‐p*‐mentha‐2,8‐dien‐1‐ol (5.32%–16.22%), *cis‐p*‐mentha‐2,8‐dien‐1‐ol (3.85%–6.39%), *trans‐p*‐mentha‐1(7),8‐dien‐2‐ol (10.45%–20.83%), and *cis‐p*‐mentha‐1(7),8‐dien‐2‐ol (12.30%–18.87%). The limonene is the only monoterpene hydrocarbon found at appreciable content (2.21%–19.28%). These results are in agreement with those obtained by Silou et al. (2006); Yang et al. (2013). According to the period of harvesting the samples, the contents of all the *p‐*menthadienol isomers are between 38% and 50%, and these contents are comparable with those of the essential oils of *Cymbopogon giganteus* from Benin (Ayedoun et al., 1999), Ivory Coast (Boti et al., 2006), and Mali (Sidibe et al., 2001). As the *Cymbopogon giganteus* and *Cymbopogon densiflorus*, it appears that *Elyonurus hensii* is an interesting species for the production of essential oil rich in *p*‐menthadienol isomers.

The essential oil obtained from the roots is rather rich in oxygenated sesquiterpenes. These compounds represent between 37.23% and 66.39% of all the essential oil, and the main sesquiterpene compound is aristolone (18%–48%). Monoterpenes hydrocarbon is also present in large quantities, they represent 13–35% of all the essential oil, and the main compound is the limonene whose yield ranges between 9% and 30% (Table 3).

**Table 3:** Chemical composition of the essential oil of the aerial part and the roots of *Elyonurus hensii* (site Loufoulakari)

Components	RI lit	RI cal	Content (%)									
			February		May		July		November		December	
			Aerial part	Roots	Aerial part	Roots	Aerial part	Roots	Aerial part	Roots	Aerial part	Roots
Tricyclene	926	922	0.82	0.34	0.78	0.22	0.47	0.10	0.35	0.35	0.89	0.30
α‐thujene	930	925	–	0.21	–	0.12	–	0.02	–	0.22	–	0.17
α‐Pinene	939	933	0.43	0.72	0.14	0.46	0.09	0.19	0.25	0.78	0.42	0.61
Camphene	954	950	2.48	1.09	2.41	0.91	1.6	0.64	1.25	1.24	2.64	0.95
Sabinene	975	973	0.07	0.19	–	0.16	–	0.06	0.05	0.21	0.05	0.15
β‐Pinene	979	980	–	–	–	–	–	–	–	–	0.21	–
Myrcene	990	989	0.29	0.54	–	0.85	–	0.45	0.48	1.05	0.04	0.51
α‐Phellandrene	1002	1005	–	0.04	–	0.02	–	–	–	–	–	0.21
*ortho* ‐Cymene	1024	1025	1.38	0.75	2.22	0.93	1.89	0.59	0.95	1.11	1.36	0.37
Limonene	1029	1029	16.99	9.73	2.21	22.8	3.21	11.4	19.28	30.4	11.98	12.22
1,8‐Cineol	1031	1032	–	3.83	0.25	3.7	0.35	1.56	–	4.51	0.16	2.39
γ‐Terpinene	1059	1057	–	0.07	–	–	–	–	–	–	–	–
Terpinolene	1088	1085	–	0.01	–	0.22	–	–	0.06	0.27	–	–
*para* ‐Cymenene	1091	1091	0.13	0.04	0.59	0.12	0.6	–	0.36	0.26	0.21	–
Linalool	1098	1101	–	–	–	0.14	–	–	–	–	–	–
Menthatriene <1.3.8‐*para*‐>	1110	1111	0.07	–	–	–	–	–	–	–	0.41	–
*Trans‐p*‐Mentha‐2,8‐dien‐1‐ol	1122	1125	10.47	1.2	6.1	1.17	5.32	0.65	16.22	2.15	9.06	1.16
Cis‐Limonene oxide	1132	1136	0.13	–	–	–	–	–	0.27	0.19	0.12	–
*Cis‐p*‐Mentha‐2,8‐dien‐1‐ol	1137	1137	5.57	0.85	4.52	0.8	3.85	0.29	6.39	1.63	6.12	0.76
*trans*‐Pinocarveol	1139	1141	0.44	–	0.81	–	0.79	–	–	–	–	–
Camphor	1146	1148	–	0.08	0.26	0.11	0.31	–	–	0.10	–	–
Pinocarvone	1164	1163	0.25	0.08	0.50	0.11	0.52	–	0.25	0.13	0.29	–
Terpinen‐4‐ol	1177	1182	0.08	0.25	–	0.62	–	0.20	–	0.5	–	–
*Trans‐p*‐Mentha‐1(7),8‐dien‐2‐ol	1189	1192	10.45	2.24	20.83	3.77	18.67	1.73	12.89	3.49	10.93	1.48
α‐Terpineol	1190	1196	–	0.47	–	0.98	–	–	–	1.22	–	0.37
3‐methyl‐Buten‐2‐al.	1197	1201	2.69	–	5.48	–	4.74	–	–	–	–	–
Caranone <*cis‐4*‐>	1200	1209	1.18	0.18	0.64	–	–	–	0.58	0.09	0.99	–
*Trans* Carveol	1229	1221	4.04	0.07	7.46	1.34	5.71	–	1.83	1.6	4.33	–
*Cis‐p*‐Mentha‐1(7),8‐dien‐2‐ol	1232	1230	12.3	2.69	18.81	2.94	16.46	1.31	13.52	3.23	12.66	1.6
*Cis*‐Carveol	1229	1233	0.69	0.82	–	–	–	–	5.36	–	0.7	0.63
Carvone	1243	1245	3.16	0.76	4.47	1.33	4.08	0.71	3.45	1.48	3.02	0.44
Piperitone	1252	1255	0.44	0.66	0.57	1.07	0.55	0.37	0.41	1.02	1.45	0.19
Perilla aldehyde	1271	1278	0.2	0.06	0.96	–	0.86	–	0.07	–	0.29	–
Phellandral	1277	1280	–	–	0.13	–	0.11	–	0.08	–	–	–
Bornyl acetate	1285	1287	0.97	0.29	1.66	0.63	1.98	0.48	1.05	0.48	1.13	0.24
Isobornyl acetate	1286	1288	0.01	–	0.17	–	0.20	–	–	–	–	–
Limonene <dioxide 2‐>	1294	1290	0.05	–	–	–	–	–	–	–	–	–
Undecanone <2‐>	1294	1292	3.13	2.63	7.75	5.86	10.14	4.33	3.52	6.2	3.44	1.97
Elemene <*beta*‐>	1390	1391	–	0.3	–	0.08	–	0.34	0.14	0.31	–	0.37
Methyl eugenol	1403	1399	–	0.32	–	0.38	–	0.31	–	0.42	–	0.19
α‐Gurjunene	1409	1412	–	–	–	0.04	–	0.18	–	0.03	–	–
Cymene 2.5‐dimethoxy‐para	1426	1413	–	0.47	–	0.59	–	0.21	–	0.52	–	0.29
Caryophyllene <E‐>	1428	1426	–	–	–	–	–	–	0.23	–	–	–
Aristola‐1(10).8‐diene	1429	1431	–	0.6	–	0.8	–	0.45	–	0.65	–	0.54
Gurjunene <beta‐>	1433	1435	–	4.59	0.07	6.30	0.13	4.16	0.14	4.71	1.07	4.10
β‐Selinene	1485	1488	–	–	–	1.13	–	–	–	0.75	–	–
Tridecan‐2‐one	1496	1495	1.32	1.88	4.53	2.71	7.12	2.11	1.97	2.60	3.10	1.67
α‐Selinene	1498	1500		0.25		0.06		0.06		0.09	–	–
α‐Bulnesene	1509	1504	–	–	–	–	–	–	–	–	–	0.28
γ‐Cadinene	1513	1516	–	0.24	–	–	–	–	–	–	–	–
δ‐Cadinene	1522	1521	–	0.07	–	0.08		–	–	–	–	–
7‐epi‐alpha‐Selinene	1524	1522	–	0.63	–	0.89	0.10	–	–	0.68	0.09	0.57
Nerolidol <E‐>	1563	1561	0.04	–	–	0.14	–	–	0.28	–	–	–
Maaliol	1567	1575	–	1.69	–	2.14	–	1.74	–	1.83	–	1.23
Spathulenol	1578	1581	–	0.28	–	0.44	–	0.41	–	0.37	–	0.29
Caryophyllene oxide	1581	1583	–	–	–	0.26	–	–	–	–	0.01	–
Rosifoliol	1600	1612	–	0.17	–	0.36	–	–	–	–	–	–
α‐Cadinol	1653	1660	–	0.17	–	0.18	–	–	–	–	–	–
Intermedeol	1666	1671	1.29	2.66	3.35	5.04	5.30	9.63	3.19	3.03	0.01	4.50
Pentadecan‐2‐one	1697	1698	0.02	0.1	0.30	0.14	–	0.17	–	0.08	2.15	0.42
Aristolone	1763	1764	–	42.23	0.31	18.30	0.60	46.9	0.60	14.1	0.98	48.11
Total			82.08	88.84	98.39	92.2	95.7	92.1	95.67	94.7	80.44	90.23

RI, retention indices.

### 3.2 Evolution of the content of the main major constituents: natural selection

**Figure 1:**
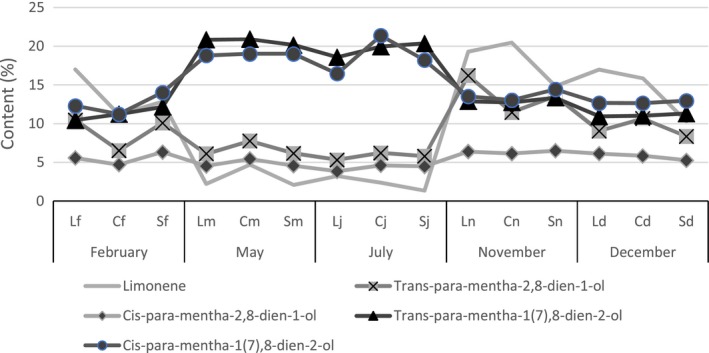
Evolution of the content of the main major constituents of the essential oil

For all three sites, Loufoulakari (L), Campus rural (C), and Sese (S), it is observed that the behavior of the five main major constituents is identical for the aerial parts. During periods of significant rainfall decreases, May (m) and July (j), the *cis* and *trans‐p*‐mentha‐1(7),8‐dien‐2‐ol isomers are produced in large quantities (on average, the content is of the order of 18%), and the content of the *trans‐p*‐mentha‐2,8‐dien‐1‐ol isomer (around of 5%) decreased in the same period for the Lm, Cm, Sm, Lj, Cj, and Sj samples (Figure 1). Moreover, for the three sites, the results show that the type of soil does not influence the production of the main major constituents. In the zone of “plateau des Cataractes” where the harvests were performed, the average temperature is 22°C (min 15°C, max 26°C) during the dry season and 28°C (min 23°C, max 33°C) during the rainy season (Samba Kimbata, 1978). It is, therefore, possible to consider that the factors related to the sunlight and to the water stress have an impact on the quantitative production of these isomers. The drastic decrease in rainfall is already an important factor influencing the yield of essential oil, and in this case, the composition of essential oil is also affected. There is a natural selection in the biosynthesis of isomers favoring the synthesis of *cis* and *trans‐p*‐mentha‐1 (7),8‐dien‐2‐ol during periods of water stress. The separation of the isomers by conventional methods of chemistry is not always easy, these results show that it just to extract the essential oil from the plant material harvested during the water stress period for obtaining extracts enriched in *cis* and *trans‐p*‐mentha‐1(7),8‐dien‐2‐ol and poor in *cis* and *trans‐p*‐mentha‐2,8‐dien‐1‐ol. This is one of the first possible steps in the separation of four isomers of *p*‐menthadienol.

**Figure 2:**
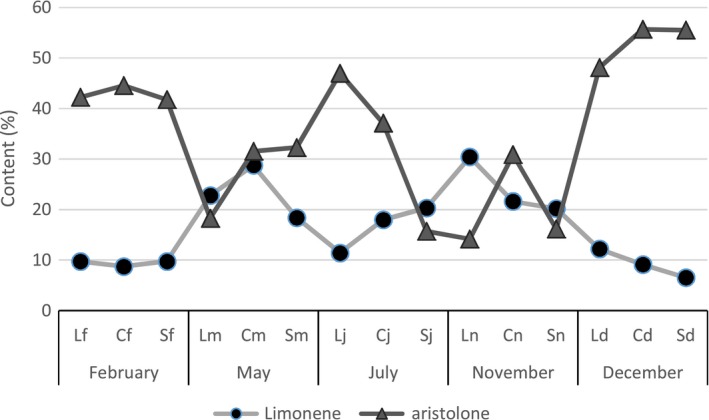
Evolution of the content of the major constituents of the roots

In the roots, on all three sites, the chemical profile is primarily aristolone. We also find a certain selectivity of the plant in the production of two major compounds. In February and December, for all three sites, the content of aristolone is at the maximum, whereas that of limonene is low. In general, it seems that the production of aristolone comes at the expense of limonene. (Figure 2).

### 3.3 Modeling of the extraction

Water distillation is used to extract essential oil. The modeling of the extraction process is in principle complex but can be simplified by the judicious choice of the factors to be studied. We selected three factors: the extraction duration (hr), X_1_; the residual water content, X_2_; and the state of division (cm), X_3_.

#### 3.3.1 Aerial part

The essential oil from the aerial part is rich in the p‐menthadienol isomers. The experimental matrix and the essential oil yields and p‐menthadienol isomers content are given in Table 4.

**Table 4:** Experimental matrix, essential oil yields, and *p‐*menthadienol isomers and aristolone contents

Run	X_1_	X_2_	X_3_	Aerial part			Roots	
				A (%)	B (%)	C (%)	D (%)	E (%)
1	−1	−1	−1	1.59	23.24	29.88	0.49	37.29
2	+1	−1	−1	2	21.89	29.77	0.91	50.02
3	−1	+1	−1	0.99	15.87	32	0.51	37.79
4	+1	+1	−1	0.83	20.39	25.11	0.83	37.30
5	−1	−1	+1	2.10	22.51	27.71	1.02	54.55
6	+1	−1	+1	2.11	18.39	28.39	0.75	36.68
7	−1	+1	+1	0.40	16.87	29.03	0.51	36.87
8	+1	+1	+1	0.65	16.66	29.04	0.91	38.34
Level ‐1	1.5	low	<5					
Level +1	3	high	10					

A: Essential oil yield; B: *Cis* and *trans‐p*‐mentha‐2,8‐ dien‐1‐ol; C: *Cis* and *trans‐p*‐mentha‐1(7),8‐dien‐2‐ol; D: Essential oil yield; E: Aristolone.

The mathematical equation representing the quantitative yield generated by the model is as follows:

$$\begin{array}{cc} Y= & 1.33+0.06x_{1}-0.61x_{2}-0.01x_{3}-0.04x_{1}x_{2}+\\ & 0.001x_{1}x_{3}-0.17x_{2}x_{3}+0.10x_{1}x_{2}x_{3} \end{array}$$

with:

$$a_{0}=1.33,\;a_{1}=0.06,\;a_{2}=-0.61,\;a_{3}=-0.01$$

$$a_{12}=-0.04,\;a_{13}=0.001,\;a_{23}=-0.17,\;a_{123}=0.10$$

The average yield of the essential oil that we can extract is *a*_0_ = 1.33% for 8 experiments on a scale ranging from 0.40% to 2.11%. In the experimental domain considered, the factors of the coefficients are low, reflecting the weak influence of factors chosen on the essential oil yield. However, there is an influence of the residual water content (*a*_2_ = −0.61) on the yield of the essential oil. The negative sign of the coefficient indicates that a high water content in the plant material does not favor the extraction of essential oil, thus confirming the observations made on modeling the extraction of the essential oils of *Eucalyptus citriodora* (Silou et al., 2009) and of *Cymbopogon citratus* (Silou et al., 2004).

Concerning the *cis* and *trans‐p*‐mentha‐2.8‐dien‐1‐ol the mathematical equation generated by the model is as follows:

$$\begin{array}{cc} Z_{a}= & 19.47-0.14x_{1}-2.03x_{2}-0.87x_{3}+\\ & 1.22x_{1}x_{2}+0.18x_{2}x_{3}-0.93x_{1}x_{3}-0.24x_{1}x_{2}x_{3} \end{array}$$

The cumulated average content of *cis* and *trans‐p*‐mentha‐2,8‐dien‐1‐ol is *a*_0 = _19.47% for all eight experiments on a scale of 15.87% to 23.24%. The effects of the main factors are negative with a large weight to the residual water content (a_2_ = −2.03): the maximum content of *cis* and *trans‐p*‐mentha‐2,8‐dien‐1‐ol is obtained when the amount of water in the plant material is low. The duration of the extraction has very little impact on the content because its coefficient factor is low. The combination of the extraction duration and the residual water content (*a*_12_ = 1.22) seems to have an important influence on the content of *cis* and *trans*‐*p*‐mentha‐2,8‐dien‐1‐ol.

The content of *cis* and *trans‐p‐*mentha‐1(7),8‐dien‐2‐ol can be modeled by the following expression:

$$\begin{array}{cc} Z_{b}= & 28.87-0.78x_{1}-0.07x_{2}-0.32x_{3}-0.93x_{1}x_{2}+\\ & 0.96x_{1}x_{3}+0.56x_{2}x_{3}+0.76x_{1}x_{2}x_{3} \end{array}$$

The cumulated average content of *cis* and *trans*‐p‐mentha 1 (7), 8‐dien‐2‐ol is 28.87% for all eight experiments on a scale of 25.11% to 32%. The coefficients of the factors are generally low: the content of *cis* and *trans*‐*p*‐mentha 1 (7), 8‐dien‐2‐ol is weakly influenced by the factors chosen. The residual water content (a_2_ = −0.07), which has a strong influence on the content of *cis* and *trans*‐*p*‐mentha‐2,8‐dien‐1‐ol, does not influence the content of *cis* and *trans*‐p‐mentha 1 (7), 8‐dien‐2‐ol. Furthermore, the combined effect of the extraction duration and the residual water content (a_12_ = −0.93) is unfavorable for the extraction of *cis* and *trans*‐p‐mentha 1 (7), 8‐dien‐2‐ol, whereas this interaction increases the content of *cis* and *trans*‐*p*‐mentha‐2,8‐dien‐1‐ol. The combined effect of the extraction duration and the state of division (a_13_ = 0.96) positively influences the content of *cis* and *trans*‐p‐mentha 1 (7), 8‐dien‐2‐ol. Finally, the residual water content is the most important influencing factor and determines the contents of *cis* and *trans*‐*p*‐mentha‐2,8‐dien‐1‐ol and *cis* and *trans*‐p‐mentha 1 (7), 8‐dien‐2‐ol.

#### 3.3.2 Roots

The essential oil yield of the roots (Table 4) is low compared to that of the aerial part. The average yield that could be extracted is 0.74% for the eight experiments, with a range of 0.49% to 1.02%.

The aristolone content of essential oil from the roots can be modeled by the following expression:

$$\begin{array}{cc} Z_{c}= & 41.10-0.52x_{1}-3.53x_{2}+0.50x_{3}+\\ & 0.76x_{1}x_{2}-3.58x_{1}x_{3}-0.47x_{2}x_{3}+4.07x_{1}x_{2}x_{3} \end{array}$$

The content of aristolone is more sensitive to the effects of the factors and their interactions. With an average content of 41.10% for eight experiments and a range of 36.68% to 54.55%, the extraction of aristolone is impacted by the residual water content with a large negative weight (a_2 = _−3.53). The effects of the interactions are also important; the combined effects of the duration of extraction and the state of division have a large negative weight (a_13 = _−3.58). Additionally, the combined effects of the duration of extraction, the residual water content and the state of division led to the most significant effect on the response (a_123 = _4.07). In the latter case, the interpretation of this interaction is very complex. However, if we consider the mathematical model in which we neglect the factors and interactions that have a minor influence, we obtain the following equation:

$$Z_{c}=41.10-3.53x_{2}-3.58x_{1}x_{3}\;4.07x_{1}x_{2}x_{3}$$

In the selected experimental area, we find that when the duration of distillation and the residual water content are low and the state of division is strong, the average content of aristolone ranges from 41.10% to 52.28%, with an increase of 11.18%. The lowest content of aristolone is obtained when the duration of the extraction and the state of division are at high levels and when the residual water content is low, which corresponds to a variation in the average content of 41.10% to 36.98% (a decrease of 4.12%). However, this interpretation must integrate the fact that the distillation time and the state of division are weak influences.

## 4 Conclusion

The essential oil of the aerial part is rich mainly in *cis* and *trans‐p*‐mentha‐2,8‐dien‐1‐ol, *cis* and *trans‐p*‐mentha‐1 (7), 8‐dien‐2‐ol. The essential oil obtained from the roots is rich in aristolone and limonene. The content of these components varies depending on the harvest period. The residual water content is the most important factor that significantly influences the average yield of the essential oil and the content of the major constituents. Regarding the aerial part, a low residual water content of the plant material increases (1) the essential oil yield and (2) the content of *cis* and *trans*‐p‐mentha‐2, 8‐dien‐1‐ol. For the roots, the samples that have a very low residual water content should provide extracts richer in aristolone. The combined effects of the extraction duration and the state of division, on the one hand, and the extraction duration, the state of division and the residual water content, on the other hand, greatly influence the extraction of aristolone. However, these interactions are complex and difficult to assess.

## Conflict of Interest

None declared.

## References

[fsn3459-bib-0001] Adams, R. P. (2012). Identification of Essential Oil Components by Gaz Chromatography/Quadrupole Mass Spectroscopy. Carol Stream, IL: Allured Publishing.

[fsn3459-bib-0002] Akhila, A. (2010). Chemistry and Biogenesis of Essential oil from the Genus Cymbopogon. In Essential Oil‐Bearing Grasses: CRC Press, Taylor and Francis Group.

[fsn3459-bib-0003] Ayedoun, M. A. , Sohounhloué, K. D. , Menut, C. , Lamaty, G. , & Bessière, J. M. (1999). Composition chimique des huiles essentielles de deux espèces de Cymbopogon du Bénin exploitable industriellement. Bioressources, Energie, Développement Environnement, 8, 4–6.

[fsn3459-bib-0004] Boelens, M. H. (1994). Sensory and chemical evaluation of tropical grass oils. Perfum Flavor, 19, 29–15.

[fsn3459-bib-0005] Boti, J. B. , Muselli, A. , Tomi, F. , Koukoua, G. , Yao Nguessam, T. , Costa, J. , & Casanova, J. (2006). Combined analysis of *Cymbopogon giganteus* Chiov. Leaf from Ivory Coast, Compte Rendus Académie des Sciences (fr). Chimie, 9, 164–168.

[fsn3459-bib-0006] Chisowa, B. H. (1997). Chemical composition of flower and leaf oils of Cymbopogon densiflorus Stapf from Zambia. Journal of Essential Oil Research, 9, 469–470.

[fsn3459-bib-0007] Clevenger, J. F. (1928). Apparatus for the determination of the volatil oil, J. Am. Pharm. Assoc., 17, 341–346.

[fsn3459-bib-0070] Davies, O. L. (1954). Design and analysis of industrial experiments. London: Olivier & Boyd.

[fsn3459-bib-0008] Denny, E. F. K . (1991). Field distillation for herbaceous oils, 2d edition. Australia: Denny & Mckenzie Associate, Lilyte.

[fsn3459-bib-0009] Goupy, J. (2001). Introduction aux plans d'expériences,2ème Edition. Paris: Dunod.

[fsn3459-bib-0010] Jirovetz, L. , Buchbauer, G. , Eller, G. , Ngassoum, M. B. , & Maponmetsem, P. M. (2007). Composition and antimicrobial activity of *Cymbopogon giganteus* (Hochst.) Chiov. essential flower, leaf and stem oils from cameroon. Journal of Essential Oil Research, 19, 385–389.

[fsn3459-bib-0011] Köning, W. A. , Hochmuth, D. H. , & Joulain, D . (2001). Terpenoids and Related Constituents of Essential Oils Library of Mass Finder 2.1 University of Hamburg, Institute of Organic Chemistry, Hamburg.

[fsn3459-bib-0012] Menut, C. , Bessiere, J. M. , Samate, D. , Djibo, A. K. , Buchbauer, G. , & Schopper, B. (2000). Aromatic Plants of Tropical West Africa. Xl. Chemical Composition, Antioxidant and Antiradical Properties of the Essential Oils of Three *Cymbopogon* Species from Burkina Faso. Journal of Essential Oil Research, 12, 207–212.

[fsn3459-bib-0013] NIST: National Institute of Standards and Technology . (2008). PC Version 1.7 of The NIST/EPA/NIH Mass Spectra Library, Perkin‐Elmer, Norwalk, CT National Institute of Standards and Technology, NIST Chemistry WebBook.

[fsn3459-bib-0014] Ortigosa, C. (1993). Planification expérimentale en chimie. Bulletin du CIFEC, 7, 46–57.

[fsn3459-bib-0015] Samba Kimbata, M. J . (1978). Le climat du Bas Congo. Thèse de 3ème cycle. Dijon, France: Univ. De Bourgogne, Centre de Recherches de Climatologie, 280 p.

[fsn3459-bib-0016] Sidibe, L. , Chalchat, J. C. , Garry, R. P. , & Hamara, M. (2001). Aromatic plants of Mali: Chemical composition of two cymbopogons: cymbopogon citratus L, Cymbopogon giganteus Chiov. L. Journal of Essential Oil Research, 13, 110–113.

[fsn3459-bib-0017] Silou, T. , Loubaki, L. , Figuérédo, G. , & Chalchat, J. C. (2006). Study of essential oil composition of *Elyonurus hensii* Schum from Congo. Journal of Essential Oil Research, 18, 518–520.

[fsn3459-bib-0018] Silou, T. , Malanda, M. , & Loubaki, L. (2004). Optimisation de l'extraction de l'huile essentielle de *Cymbopogan citratus* grâce à un plan factoriel complet 2^3^ . Journal of Food Engineering, 65, 2199–2223.

[fsn3459-bib-0019] Silou, T. , Mapola, G. , Makany, R. A. , Loumouamou, A. N. , Malanda, M. , & Chalchat, J. C . (2009). Model of steam and water extraction of Essential oil of Eucalyptus Citriodora using a complete 2^n^ factorial plan. International Journal of Food Engineering, 5(4), Art. 9.

[fsn3459-bib-0020] Tian‐Shung, W. , Damu, A. G. , Su, C. –R. , & Kuo, P.‐C. (2004). Terpenoids of Aristolochia and their biological activities. Natural Products Reports, 21, 594–624. 10.1039/b401950d 15459757

[fsn3459-bib-0021] Van Del Dool, H. , & Kratz, P. D. (1963). A generalization of the retention index system including linear temperature programmed gas‐liquid partition chromatography. Journal of Chromatography A, 11, 463–471. 10.1016/s0021-9673(01)80947-x 14062605

[fsn3459-bib-0022] Yang, Y. , De Cian, M. C. , Nsikabaka, S. , Tomi, P. , Silou, T. , Costa, J. , & Paolini, J. (2013). Volatile Fraction Composition and Total Phenolic and Flavonoid Contents of *Elionurus hensii*‐Antioxidant Activities of Essential Oils and Solvent Extracts. Natural Product Communications, 8(5), 655–661.

